# Biomimetic Effect of Nano-Hydroxyapatite in Demineralized Enamel before Orthodontic Bonding of Brackets and Attachments: Visual, Adhesion Strength, and Hardness in In Vitro Tests

**DOI:** 10.1155/2020/6747498

**Published:** 2020-01-30

**Authors:** Andrea Scribante, Mohammad Reza Dermenaki Farahani, Giorgio Marino, Claudia Matera, Ruggero Rodriguez y Baena, Valentina Lanteri, Andrea Butera

**Affiliations:** ^1^Unit of Orthodontics and Pediatric Dentistry, Section of Dentistry, Department of Clinical, Surgical, Diagnostic and Pediatric Sciences, University of Pavia, Pavia, Italy; ^2^Unit of Dental Hygiene, Section of Dentistry, Department of Clinical, Surgical, Diagnostic and Pediatric Sciences, University of Pavia, Pavia, Italy; ^3^Department of Biomedical, Surgical and Dental Science, University of Milan, Milan, Italy

## Abstract

Dietary habits with high consumption of acidic food can induce in orthodontic patients an increased risk of demineralization lesions around orthodontic brackets and bands. The purpose of the present laboratory study is to assess the *in vitro* visual efficacy of a biomimetic nano-hydroxyapatite remineralizing solution in a hypomineralized enamel surface and its effect on adhesion of fixed orthodontic appliances and on enamel microhardness. Intact teeth were demineralized, and subsequently the areas of demineralization were visually recorded using a 0–100 scale. Subsequently, a remineralizing solution (Biorepair® Repair Shock Treatment) was applied for ten minutes once a day/for one week per month for a total remineralizing treatment of 3 months. Visual effects were recorded. Moreover, bond strength was recorded and adhesive remnant index scores were measured for both orthodontic brackets and composite attachments both before demineralization and after demineralization and application of remineralizing solution. Also, Vickers microhardness was measured. All data were submitted to statistical analysis. The application of remineralizing solution induced a significant *in vitro* reduction of demineralized areas after the first week of application. No significant differences between untreated enamel surfaces and remineralized surfaces were detected after 2 months of remineralizing treatment. Bond strength values were significantly reduced for both brackets and attachments after remineralizing treatment. However, attachments showed higher adhesion values than brackets in both conditions tested. Remineralized enamel showed significantly higher microhardness values than demineralized enamel and lower values than intact enamel.

## 1. Introduction

Dental erosion is a process consisting in the loss of hard substance of the tooth as a consequence of frequent and prolonged exposure to acidic agents [1]. Assuming that the solubility of hydroxyapatite is low (pH of 5.5), it tends to increase as the oral pH decreases [2].

The prevalence of dental erosion presents very variable results in different areas of the world. In Europe, the data indicate that around 30% of the population aged between 18 and 35 has at least one tooth affected by an erosive process [3].

Diffusion and entity of the problem change in different individuals because of the presence of predisposing factors of endogenous and exogenous origin, such as acid salivary pH, diet rich in acidic foods and low in calcium and phosphates, disorders related to the gastrointestinal tract (e.g., gastroesophageal reflux and gastritis), eating disorders associated with vomiting, such as bulimia, insufficient oral hygiene level, and presence of multibracket orthodontic therapy that predispose to prolonged contact with the acid action deriving from the bacterial metabolism of the biofilm [4].

During orthodontic treatment, the presence of the appliance can influence periodontal parameters [5] and raise the risk of dental erosion [6]. The management of dental enamel involves both correct oral hygiene practices and the application of remineralizing products, based on calcium, phosphates, and fluorides [7].

Domestic and professional oral hygiene techniques both aim to scrupulous removal of the biofilm. On the contrary, the use of fluoride-based products has always been the gold standard for the remineralization of hard tissues, thanks to its ability to integrate into the enamel transforming the hydroxyapatite in fluorapatite [8].

However, recent research has moved towards the introduction of new remineralizing techniques alternative to fluoride and based on the integration of calcium and phosphates at the level of demineralized dental surfaces [9].

Therefore, the objective of the present report was to assess an *in vitro* preliminary test of visual efficacy of a remineralizing solution in a hypomineralized enamel surface and its effect on adhesion of fixed orthodontic appliances and on enamel microhardness. The first null hypothesis of the present research was that there is no difference in visual remineralization effect between test and control groups. Additionally, the second null hypothesis of the present study was that there is no difference between brackets and attachments bonded onto intact enamel and after hypomineralization and remineralization treatment. Finally, the third null hypothesis was that there is no significant difference in microhardness values among various groups.

## 2. Materials and Methods

The Internal Unit Institutional Committee Board approved the protocol of the study. Both visual mineralization and adhesion tests were performed.

### 2.1. Visual Remineralization Effect

Sample size calculation was conducted taking into consideration of previous studies about visual evaluation on a grid-based scale. The present report evaluated continuous endpoint. The confidence level was set at 95% (alpha error 0.05) and 80% power. For each of the 2 groups, 10 tests were needed.

20 freshly extracted bovine mandibular incisors were collected. For the selection, the following inclusion criteria were followed: integrity of the buccal and lingual surfaces, absence of enamel wear, absence of caries, absence of volume, shape and structure anomalies, and absence of traumatic lesions [10]. The selected teeth were stored in thymol solution (0.1% weight/volume) [11]. All the elements were numbered and photographed (t0) with an optical microscope (Stereomicroscope SR, Zeiss, Oberkochen, Germany).

A 25 mm^2^ area (5 mm length and 5 mm height) of the vestibular surfaces of the elements was conditioned with 37% orthophosphoric acid (orthophosphoric acid gel, 3M Unitek, Monrovia, California, USA), left to act for 2 hours in order to obtain a radical demineralization [12]. Subsequently, the teeth were rinsed by using a spray of water. Then, the specimens were dried for 10 seconds. Photographs were retaken (t1) with the optical microscope to assess the demineralization obtained, and then the teeth were stored in physiologic solution and randomly divided in two groups: a control group that remained demineralized and a test group that underwent remineralizing treatment.

The recently introduced biomimetic hydroxyapatite-based remineralizing solution that has been tested in the present report (Biorepair® Repair Shock Treatment, Coswell, S.p.A., Funo, Italy) contains the following ingredients: aqua, zinc hydroxyapatite 30%, hydrated silica, silica, sodium myristoyl sarcosinate, sodium methyl cocoyl taurate, sodium bicarbonate, aroma, sodium saccharin, phenoxyethanol, benzyl alcohol, sodium benzoate, citric acid, and menthol.

Biomimetic effect of the nano-hydroxyapatite is based on its interaction with biological tissues and on its ability to mimic biogenic materials in their functionalities. Chemical composition is similar to enamel and dentin, with intermediate characteristics. Gradual action of biomimetic nano-hydroxyapatite crystals allows the linkage to dentinal and enamel tooth surfaces due to bio-reabsorption properties under physiological conditions. This property can be modulated by ion substitution and crystallinity degree achieved implementing innovative synthesis with nanosized crystal control [1].

For test group teeth, the vestibular surfaces were treated with 3 remineralizing cycles through the biomimetic hydroxyapatite-based remineralizing solution. Each cycle consisted of applying the product for 10 minutes a day for one week in a month. At the end of each cycle, the surfaces were photographed again with an optical microscope, and the teeth were stored in physiologic solution between one cycle and the other.

**Figure 1:**
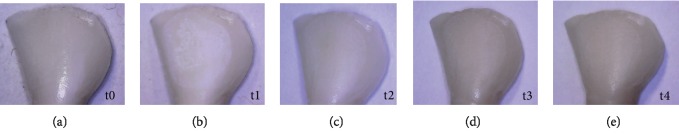
Microphotographs of a specimen during the different times. Freshly extracted tooth (t0); sample after demineralization (t1); sample after first remineralization cycle (t2); sample after second remineralization cycle (t3); sample after third remineralization cycle (t4).

All the specimens were photographed (Figure 1) with an optical microscope (Stereomicroscope SR, Zeiss, Oberkochen, Germany) to register changes in mineralization at different times: 1 month after demineralization (t2); 2 months after demineralization (t3); and 3 months after demineralization (t3).

The lingual surfaces of the teeth were also etched with 37% orthophosphoric acid left to act for 2 hours, washed, and dried thoroughly, without any subsequent additional treatment, to act as the control group. The surfaces were photographed with an optical microscope with the same timing as the test group.

Demineralization was visually inspected with a graduated 100-slot grid superimposed to each microphotograph in order to assess the percentage of hypomineralization for each area. A score was allocated to each specimen, thus indicating the percentage of visual demineralized area.

### 2.2. Adhesion Test

Sample size calculation was conducted taking into consideration of previous reports about other remineralizing agents, as in the literature, and there are no studies about biomimetic nano-hydroxyapatite. The present research evaluated a continuous endpoint. The confidence level was set at 95% (alpha error 0.05) and 80% power. For each of the 6 groups, 10 tests were needed.

60 freshly extracted mandibular bovine incisors were collected. For the selection, the following inclusion criteria were followed: integrity of the buccal and lingual surfaces, absence of enamel wear, absence of caries, absence of volume, shape and structure anomalies, and absence of traumatic lesions [10]. The teeth were stored in thymol (0.1% weight/volume) [13].

Subsequently, the teeth were randomly divided into 6 groups of 10 elements each:

 
*Group 1*. 10 stainless steel MBT orthodontic brackets (Victory, 3M, Monrovia, CA, USA) were bonded onto intact enamel with an orthodontic adhesive (Transbond XT® Primer and resin, 3M, Glendora, USA) following the manufacturer's instruction.
 
*Group 2*. 10 stainless steel MBT orthodontic brackets (Victory, 3M) were bonded with an orthodontic adhesive (Transbond XT® Primer and resin, 3M) onto enamel that was previously demineralized with 37% orthophosphoric acid (orthophosphoric acid gel, 3M Unitek, Monrovia, California, USA), left to act for 2 hours in order to obtain a radical demineralization.
 
*Group 3*. 10 stainless steel MBT orthodontic brackets (Victory, 3M) were bonded with an orthodontic adhesive (Transbond XT® Primer and resin, 3M) onto enamel that was previously demineralized with 37% orthophosphoric acid (orthophosphoric acid gel, 3M Unitek, Monrovia, California, USA), left to act for 2 hours in order to obtain a radical demineralization and subsequently remineralized. The vestibular surfaces were treated with 3 remineralizing cycles through a biomimetic hydroxyapatite-based remineralizing solution (Biorepair® Repair Shock Treatment, Coswell). Each cycle consisted of applying the product for 10 minutes a day for 7 days a month. At the end of each cycle, the teeth were stored in physiologic solution between one cycle and the other.
 
*Group 4*. 10 attachments were prepared using a silicon mold filled with composite resin (Filtek Supreme XTE® Flow Shade A2, 3M, Glendora, USA) and then bonded onto intact enamel with an orthodontic adhesive (Transbond XT® Primer and resin, 3M).
 
*Group 5*. 10 attachments were prepared using a silicon mold filled with composite resin (Filtek Supreme XTE® Flow Shade A2, 3M) and then bonded with an orthodontic adhesive system (Transbond XT® Primer and resin, 3M) onto enamel that was previously demineralized with 37% orthophosphoric acid (orthophosphoric acid gel, 3M Unitek), left to act for 2 hours in order to obtain a radical demineralization.
 
*Group 6*. 10 attachments were prepared using a silicon mold filled with composite resin (Filtek Supreme XTE® Flow Shade A2, 3M) and then bonded with an orthodontic adhesive system (Transbond XT® Primer and resin, 3M) onto enamel that was previously demineralized with 37% orthophosphoric acid (orthophosphoric acid gel, 3M Unitek), left to act for 2 hours in order to obtain a radical demineralization and subsequently remineralized using the same protocol of Group 3.

Teeth of all groups were embedded into resin blocks, so their vestibular surface was parallel to shearing force [14]. Both brackets (Groups 1 and 3) and attachments (Group 2 and 4) were tested with a universal testing machine (Model 3343, Instron, Canton, MA, USA) in order to register the shear bond strength. Each appliance was stressed vertically (with an occluso-gingival direction and a speed of the crosshead set at 1 mm/min) [15]. The load required to detach the brackets was measured in newton and then converted into megapascal (MPa) [13].

After microscopy examination (Stereomicroscope SR, Zeiss) at x20 magnification, the adhesive remnant index (ARI) score was measured to evaluate the amount of adhesive left on the enamel after debonding procedure [16]. The ARI scale ranges from 0 to 3 as a function of resin amount left in the tooth (0: no resin remaining on the tooth; 1: less than 50% resin remaining on the tooth; 2: more than 50% resin remaining on the tooth; 3: 100% resin remaining on the tooth).

### 2.3. Microhardness Test

As in previous reports that evaluated the microhardness of dental materials [17–20], each specimen's microhardness was determined with a microhardness tester (Isoscan HV2, LTF Spa, Antegnate, Bergamo, Italy) using a Vickers diamond indenter. Three indentations were made equally placed over a circle, each being no closer than 0.5 mm to the adjacent indentation, using a 25 g load with a 5 s dwell time [21]. The two diagonal lengths of each indentation were measured by a 40x magnification built-in scale microscope and were converted into a microhardness value (VHN) using the following equation: HV = 1.854 P/d2, where HV is microhardness in kg/mm^2^, P is the load in kgf, and *d* is the average length of the diagonals in mm [22]. For a given specimen, the three hardness values for each surface were averaged and reported as a single value [23].

### 2.4. Statistical Analysis

Computer software (R® version 3.1.3, R Development Core Team, R Foundation for Statistical Computing, Wien, Austria) was used to perform statistical analysis. Descriptive statistics for all groups included mean, standard deviation, minimum, median, and maximum values. The KolmogorovSmirnov test assessed normality of distributions.

Analysis of variance (ANOVA) for repeated measures and Tukey tests were used for inferential statistics of visual hypomineralization values. Analysis of variance (ANOVA) and Tukey tests were applied for inferential statistics of bond strength values and microhardness measurements. The chi-square test was used for ARI score distributions.

Significance was predetermined at *P* < 0.05 for all statistical tests.

## 3. Results

**Figure 2:**
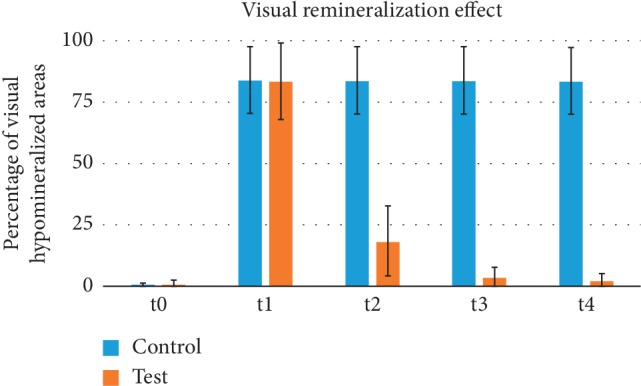
Percentage of visual hypomineralized areas for control and experimental groups (mean and standard deviation). Before acid treatment (t0); after acid treatment (t1); 30 days after acid treatment (t2); 60 days after acid treatment (t3); 90 days after acid treatment (t4).

The results of visual hypomineralization areas are reported in Figure 2.

As shown in Table 1, for control group, at t0 all teeth presented no hypomineralized areas. After acid exposure (t1), all the tested elements showed a significant increase in hypomineralized area count (*P* < 0.0001). No significant differences in hypomineralized areas were detected (*P* > 0.05) among the other times tested (t1, t2, t3, and t4).

For the test group, at t0 all teeth presented no hypomineralized areas. After acid exposure (t1), teeth showed a significant increase in hypomineralized area count (*P* < 0.0001). After the first cycle of remineralizing treatment (t2), a significant reduction in hypomineralized area count was reported (*P* < 0.0001). After the second cycle of remineralizing treatment (t3), an additional significant reduction in hypomineralized area count was reported (*P* < 0.05). After the third cycle of remineralizing treatment (t4), no further significant difference was reported (*P* > 0.05).

The results of the shear bond strength values are reported in Table 2.

The descriptive statistics for the shear bond strength (MPa) of the 6 groups tested are presented in Table 2. Significant differences were detected after the ANOVA test (*P* < 0.0001). Post-hoc analysis with the Tukey test reported that the highest shear bond strength values (*P* < 0.05) were reported when appliances were bonded on intact enamel (Groups 1 and 4). Significantly lower values (*P* < 0.05) were registered when enamel was demineralized and remineralized (Groups 3 and 6). The lowest (*P* < 0.05) adhesion values were noticed after demineralization (Groups 2 and 5). Moreover, brackets and attachments reported no significant differences between them, in all the conditions tested (*P* > 0.05).

ARI scores are reported in Table 3. The chi-squared test showed significant differences among various groups in their frequency distributions (Table 3). For control groups, a significantly higher frequency of high ARI scores (ARI = 2 and ARI = 3) was reported. Conversely, after enamel demineralization and after demineralization and remineralization, a significant frequency of ARI scores of “0” and “1” was reported (*P* < 0.05). No significant differences in ARI score distribution were reported between brackets and attachments when comparing control groups (1 and 3) demineralized enamel groups (2 and 4) and demineralized and remineralized groups (3 and 6) (*P* > 0.05).

The results of the microhardness test are reported in Table 4. The highest values were reported on intact enamel. Significantly lower values were reported with demineralized and remineralized enamel (*P* < 0.05). The lowest values were reported with demineralized enamel (*P* < 0.05).

## 4. Discussion

First, second, and third null hypotheses of the present research were rejected.

The results showed that after the first cycle of remineralizing treatment (t2), a significant reduction in visual hypomineralized area count was reported in the test group. Moreover, after the second cycle of remineralizing treatment (t3), an additional significant reduction in hypomineralized area count was reported. After the third cycle of remineralizing treatment (t4), no further significant difference was reported. Therefore, the remineralizing solution tested seems to be able to reduce hypomineralized areas *in vitro*.

In the literature, many techniques and materials have been investigated in order to test remineralizing agents both on dentine [24] and enamel [25]. Most studies explored fluoride-based varnishes [26] or casein phosphopeptide-amorphous calcium phosphate pastes [27]. These active ingredients have been tested for individual or combined use [28].

Nowadays, there are no studies in the literature that tested biomimetic nano-hydroxyapatite and reduction of enamel hypomineralization. The remineralization process is linked to the ability of the biomimetic hydroxyapatite to integrate completely within the enamel structure, such as physiological hydroxyapatite. This product is characterized by high concentration (30%) of microrepair (nano-hydroxyapatite crystals), more than other equivalent products, and absence of fluoride, SLS, silica abrasives, titanium dioxide, and parabens. Moreover, this product has the ability to repair enamel microscale specimens protecting from hypersensitivity, erosions, wear, and caries [29].

Biomimetic nano-hydroxyapatite is synthesized in a bulk Ca/P molar ratio of 1.7. Moreover, it contains about 4 ± 1 wt% of carbonate ions. These ions replace especially phosphate groups. Biomimetic carbonate–hydroxyapatite nanocrystals can aggregate in clusters. These aggregates present a nanostructured surface area of about 80 m^2^/g. These crystals have been reported to be used in toothpastes and mouthwashes as mineralized agents [30].

The consistency of nano-hydroxyapatite solution is not toothpaste-like, but it is a solid mousse material to be applied on tooth surfaces following normal brushing. As per the manufacturer's instructions, it reaches its greatest effectiveness if applied daily for a minimum of 2‐3 minutes up to a maximum of 10 minutes for 10 days a month. The treatment can be repeated cyclically several times a year, depending on the patient's needs.

The present study showed that a great visual remineralization effect can be noticed already after the second application cycle of biomimetic nano-hydroxyapatite, as no significant visual differences were reported between control and test groups at t3.

The remineralizing properties of biomimetic nano-hydroxyapatite are particularly useful for patients with high risk of enamel demineralization, which can be linked with greater possibility of carious lesions [31]. In fact, this treatment could be useful in patients with higher caries risk, such as orthodontic patients. In fact, the positioning of orthodontic brackets (for conventional orthodontic treatment) or attachments (for application of transparent aligner devices) onto enamel can be related with higher demineralization and caries risk [32]. For this reason, in this report, the application of biomimetic nano-hydroxyapatite has been tested before bonding brackets and attachments in order to test its effects on adhesion of orthodontic devices.

In our study, the adhesion test was performed onto intact enamel, demineralized enamel, and remineralized enamel after hypomineralization. Previous authors performed the bond strength test onto demineralized enamel demonstrated that demineralization significantly reduces shear bond strength values of enamel [33]. This is in agreement with the present report. The present report showed that shear bond strength values after remineralizing treatment were higher than demineralized surfaces but lower than those measured on intact enamel.

To our knowledge, in the literature, there are no reports that measured shear bond strength of orthodontic appliances after application of biomimetic nano-hydroxyapatite. Previous studies evaluated adhesion values after application of tricalcium phosphate [33], fluoride [34], and casein phosphopeptide-amorphous calcium phosphate [35] as remineralizing agents, used as pretreatment or incorporated into the adhesive system.

Higher bond values were reported when comparing remineralized enamel with demineralized enamel [33]. However, when remineralizing agents were compared with normal enamel, similar [35] or lower [36] bond strength values were reported.

In our study, the mean shear bond strength values ranged between 20 and 27 MPa for intact enamel, between 4 to 6 MPa for demineralized enamel, and between 12 to 19 MPa for remineralized enamel after demineralization. Concerning adhesion force values, in the literature, nowadays there are no guidelines and the force limits have not been defined clearly. Generally, to obtain sufficient adhesion, a minimum adhesion strength of 5–10 MPa is needed to sustain the masticatory forces. At the same time, bonding forces should not be too high in order to avoid enamel loss after appliance debonding (40–50 MPa) [37, 38]. Therefore, an ideal orthodontic biomaterial would have shear bond strength values not exceeding nor lowering these limits, with an amount included between 5 and 50 MPa [13]. The results of the present report respect these limitations when testing intact and remineralized enamel; therefore, these groups (1, 3, 4, and 6) present clinically acceptable bond strength values. On the contrary, when demineralization was not followed by the remineralizing agent (Groups 2 and 5), the bond strength values were borderline with the lower limit, thus supposing higher appliance failure under masticatory forces.

In the present report, teeth have been stored in thymol as in previous *in vitro* studies [10, 11, 14]. As saliva can act as a remineralizing agent, also this variable would be considered; therefore, changing storage media (artificial saliva, water, etc.), the results could vary in terms of remineralization and bond strength values.

Concerning ARI scores, the present report showed lower ARI scores both after demineralization and after remineralizing treatment. In fact, both brackets and attachments showed a significantly higher frequency of ARI = 2 and ARI = 3 when bonded onto normal enamel and a higher frequency of ARI = 0 and ARI = 1 when bonded onto demineralized enamel and onto demineralized and subsequently remineralized enamel. In fact, both demineralization and the remineralization treatment tested seem to influence ARI scores. There are no advantages directly related to high or low ARI scores. In fact, ARI = 0 could imply higher resin bonding to the appliance base than to the tooth surface after debonding. Some authors supposed that in this case less time is needed for adhesive removal from enamel. However, lower adhesion strength is expected [39]. On the contrary, ARI = 3 indicates failure between the appliance and the adhesive. This particular condition is expected to lower the risk of enamel fracture during debonding procedures [40].

Previous studies in the literature evaluated ARI scores after remineralizing agent application, showing conflicting results, with ARI score prevalence ranging from minimum to maximum values [33, 35, 36, 41]. However, a direct comparison with the results of the present report is difficult, as biomimetic nano-hydroxyapatite has not been tested yet.

The present report demonstrated that the highest microhardness values were reported with intact enamel (327.43 VHN). Lowest values were reported for demineralized enamel (238.76 VHN). These values are in agreement with previous reports that tested microhardness of intact and demineralized enamel [17–21]. Additionally, the present report tested also enamel after demineralization followed by remineralization treatment, showing intermediate values (278.97 VHN). To our knowledge, only few authors evaluated microhardness of enamel remineralized with nano-hydroxyapatite serum [19] showing similar values. Previous studies evaluated other remineralizing agents such as fluoride varnish [18] or amorphous calcium phosphate [17] and showed a partial increase of microhardness values after remineralizing treatments, as in our study.

The present preliminary report evaluated microscopic observation for remineralized teeth, bracket bond strength, and enamel microhardness. It would be useful in further investigations to chemically analyze the remineralization, in order to complete the results of the present initial evaluation of this extremely novel and untested material. Additionally, the recently introduced biomimetic hydroxyapatite-based remineralizing solution that has been tested in the present report contained zinc hydroxyapatite 30%. This percentage has not been tested yet as is the highest ever produced. In fact, in the market, other two concentrations are currently available (20% and 22%). Considering also this variable, future reports could deepen the present results, for example, adjusting remineralizing solution with different concentrations or different compositions and then testing the remineralization behavior considering also these variables.

The main limitations of the present report are related to the fact that the remineralization effect was assessed with a visual scale, and in future, a SEM evaluation could be useful to confirm the visual remineralization effect. Moreover, the adhesion values have been calculated *in vitro*, and further clinical research is needed to prove the efficacy of the product also *in viv*o. Additionally, the present preliminary study tested a single commercial product, and it would be useful to compare these preliminary results with other remineralizing agents in future investigations.

Finally, it would interesting to subject the specimens to further tests in order to evaluate the results after cyclic dynamic load and thermo cycling process, as results could partially change introducing these variables.

Based on the results of the present study and within its limits, the use of biomimetic nano-hydroxyapatite could be recommended in demineralized surfaces either in patients with no orthodontic treatment and during orthodontic treatment (when brackets and attachments have already been bonded). On the contrary, it would be better to avoid the total remineralization treatment immediately before bonding, as it could lower shear bond strength. Delaying for a couple of months or the reduction of remineralizing treatment to only one application could be recommended in order to reduce appliance failure risk [42], even if these specific conditions have not been tested yet with biomimetic nano-hydroxyapatite, and further research is needed about this new interesting topic. Moreover, the external validity of the present preliminary study should taken into careful consideration, since being an *in vitro* study using animal teeth, extrapolation should be performed with caution as biological behavior could be different.

In fact, during orthodontic patients care, biomimetic materials could be very useful for reducing demineralization of hard tissues during active treatment. However, also the health of soft tissues has to be taken into careful consideration, in order to reduce the risk of gingivitis and periodontal disease. Some in office or domiciliary therapies have been proposed during last years. Therefore, air polishing deplaquing and ozonized water treatment could be considered and matched with biomimetic protocol for a complete prevention reducing both plaque index and bleeding score [43, 44].

## 5. Conclusions

The present study demonstrates the following:

- The use of biomimetic nano-hydroxyapatite induced a visible enamel remineralization after 2 cycles of application.
- Concerning both bond strength and microhardness tests, highest values were recorded on intact enamel. Lowest strengths were reported after demineralization. Intermediate values were reported when demineralized enamel was submitted to remineralizing treatment using biomimetic nano-hydroxyapatite. Adhesion values can be considered clinically acceptable.
- Highest ARI scores were recorded for intact enamel. Lower values were scored both after demineralization and after remineralization.

## Figures and Tables

**Table 1:** Descriptive statistics of percentage of visual hypomineralized areas for control and experimental groups: before acid treatment (t0); after acid treatment (t1); 30 days after acid treatment (t2); 60 days after acid treatment (t3); and 90 days after acid treatment (t4).

Group	Time	Mean	SD	Min	Median	Max	Tukey^*∗*^
Control	t0	0.32	0.75	0.00	0.00	3.00	A
Control	t1	84.16	13.52	55.00	83.00	100.00	B
Control	t2	84.00	13.57	55.00	83.00	100.00	B
Control	t3	83.95	13.59	55.00	83.00	100.00	B
Control	t4	83.79	13.66	55.00	83.00	100.00	B
Test	t0	0.53	1.17	0.00	0.00	4.00	A
Test	t1	83.68	15.56	54.00	88.00	100.00	B
Test	t2	18.37	14.21	0.00	19.00	54.00	C
Test	t3	3.61	4.23	0.00	2.00	15.00	A
Test	t4	2.00	3.43	0.00	0.00	10.00	A

^*∗*^Tukey grouping-means with the same letters are not significantly different.

**Table 2:** Adhesion values of the six groups tested. Shear bond strength has been measured in MPa (conditions: untreated enamel, enamel after hypomineralization treatment, and enamel after hypomineralization and remineralization treatments).

Group	Appliance	Enamel	Mean	St Dev	Min	Mdn	Max	Significance^*∗*^
1	Bracket	Intact	20.73	8.97	10.02	18.78	37.87	A, D
2	Bracket	Demineralized	4.68	1.38	2.67	4.52	7.54	B
3	Bracket	Demineralized and remineralized	12.31	3.91	6.98	12.03	20.41	C
4	Attachment	Intact	27.82	3.61	20.09	28.08	34.12	A
5	Attachment	Demineralized	6.35	1.54	3.09	7.04	7.70	B
6	Attachment	Demineralized and remineralized	19.77	7.23	13.63	15.66	34.02	C, D

^*∗*^Tukey grouping-means with the same letters are not significantly different.

**Table 3:** ARI score percentages of the six groups tested (conditions: untreated enamel, enamel after hypomineralization treatment, and enamel after hypomineralization and remineralization treatments).

Group	Appliance	Enamel	ARI = 0	ARI = 1	ARI = 2	ARI = 3
1	Bracket	Intact	0	10	50	40
2	Bracket	Demineralized	100	0	0	0
3	Bracket	Demineralized and remineralized	70	30	0	0
4	Attachment	Intact	10	10	60	20
5	Attachment	Demineralized	80	20	0	0
6	Attachment	Demineralized and remineralized	60	20	0	20

**Table 4:** Descriptive statistics of microhardness values (VHN) in the three different conditions tested (untreated enamel, enamel after hypomineralization treatment, and enamel after hypomineralization and remineralization treatments).

Group	Enamel	Mean	St Dev	Min	Mdn	Max	Significance^*∗*^
1	Intact	327.43	41.22	262.65	320.00	386.90	A
2	Demineralized	238.76	33.85	198.85	229.22	297.86	B
3	Demineralized and remineralized	278.97	29.54	215.82	288.99	308.46	C

## Data Availability

All data are available upon request to the corresponding author.
